# Inpatient mortality of HIV-infected adults in sub-Saharan Africa and possible interventions: a mixed methods review

**DOI:** 10.1186/s12913-014-0627-9

**Published:** 2014-12-03

**Authors:** Bahati MK Wajanga, Lauren E Webster, Robert N Peck, Jennifer A Downs, Kedar Mate, Luke R Smart, Daniel W Fitzgerald

**Affiliations:** Department of Internal Medicine, Bugando Medical Centre, Bugando Hill Road, Box 1370, Mwanza, Tanzania; Department of Internal Medicine, Catholic University of Health and Allied Sciences, Bugando, Mwanza Tanzania; Weill Cornell Medical College, New York, NY USA

**Keywords:** HIV, AIDS, Sub-Saharan Africa, Mortality, Health system, Inpatients

## Abstract

**Background:**

Despite the increased availability of anti-retroviral therapy, in-hospital HIV mortality remains high in sub-Saharan Africa. Reports from Senegal, Malawi, and Tanzania show rates of in-hospital, HIV-related mortality ranging from 24.2% to 44%. This mixed methods review explored the potential causes of preventable in-hospital mortality associated with HIV infections in sub-Saharan Africa in the anti-retroviral era.

**Results:**

Based on our experience as healthcare providers in Africa and a review of the literature we identified 5 health systems failures which may cause preventable in-hospital mortality, including: 1) late presentation of HIV cases, 2) low rates of in-hospital HIV testing, 3) poor laboratory capacity which limits CD4 T-cell testing and the diagnosis of opportunistic infections, 4) delay in initiation of anti-retroviral therapy in-hospital, and 5) problems associated with loss to follow-up upon discharge from hospital.

**Conclusion:**

Our findings, together with the current available literature, should be used to develop practical interventions that can be implemented to reduce in-hospital mortality.

## Introduction

The aim of the literature review is to explore the potential causes of preventable in-hospital mortality associated with HIV infections in sub-Saharan Africa in the anti-retroviral era, and to discuss possible health systems improvements that may reduce this mortality. This review focuses on the in-hospital deaths that could be prevented with the current knowledge and resources available in many hospitals in sub-Saharan Africa. We recognize that a substantial number of HIV-related deaths in sub-Saharan Africa occur among community dwelling individuals who do not have access to care and medications. However, we are specifically interested in deaths in areas where HIV testing, opportunistic infection (OI) treatment, and anti-retroviral treatment (ART) are available, and yet breakdowns in the health system lead to AIDS-related deaths.

Studies in sub-Saharan Africa have documented the high rates of in-hospital fatality due to HIV. The Infectious Diseases Clinic of Fann Teaching Hospital in Dakar, Senegal, saw an in-hospital fatality rate of 44% among HIV-positive admitted individuals [1]. In 2004, Kamuzu Central Hospital (KCH) in Malawi gained access to free ART and yet in 2008 and 2009, a study found the mortality rate of HIV-positive individuals admitted to KCH was 24.2% [2]. Despite these high levels of in-hospital mortality, little information currently exists on causes of in-hospital HIV deaths in sub-Saharan Africa and how to prevent them.

Our own experience at Bugando Medical Centre has been similar. Bugando is a tertiary referral and teaching hospital that serves the Lake Victoria region of northwestern Tanzania (population: 13 million). BMC is located in the city of Mwanza, the second largest urban center in Tanzania and the capital of the Mwanza region. BMC has 100 adult medical beds located in four wards: two male and two female. The HIV prevalence in the Mwanza region is approximately 6%, and ART has been provided free of charge at BMC since 2007. Despite the availability of ART, HIV still accounts for 22% of all of our medical admissions and 33% of all deaths on the medical wards, and the in-hospital mortality rate for adults admitted with HIV is 28.6% [3].

Health care delivery systems in sub-Saharan Africa still face numerous challenges in decreasing HIV/AIDS mortality despite the increase in availability of ART for those who are eligible. In June 2001, a United Nations General Assembly Special Session (UNGASS) on HIV/AIDS was held in New York with the goal of uniting countries to address the problem of HIV/AIDS. The assembly recognized the urgency of the epidemic and secured a global commitment to universal access to HIV/AIDS care [4]. The Global Fund and the US President’s Emergency Plan for AIDS Relief (PEPFAR) were soon created. The Global Fund became a major financer of programs that fight HIV/AIDS, tuberculosis, and malaria, and PEPFAR provided increased funding in sub-Saharan Africa for the roll-out of HIV services including anti-retroviral therapy [5].

There are nearly 34 million people worldwide with HIV, of which, approximately 22.5 million reside in sub-Saharan Africa [6,7]. In 2011, about 6.2 million people in sub-Saharan Africa had access to antiretroviral therapy – that is slightly over half of the individuals who are eligible and an increase of over 1 million individuals compared to the previous year [5]. For those with access to ART, HIV no longer needs to be a death sentence. A recent study showed that life expectancy of HIV-positive individuals in the US who begin ART approaches the life expectancy of HIV-negative individuals [8]. The efficacy of combination ART has improved, and access to ART has increased drastically; therefore, in many settings drug efficacy and lack of drugs can no longer be blamed for the high rates of in-hospital mortality.

**Figure 1:**
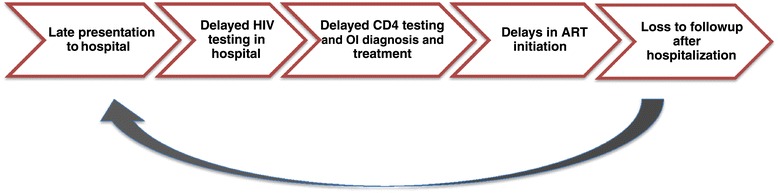
**Potential health systems failures leading to preventable in-hospital HIV/AIDS mortality in Africa.**

Therefore, we conducted a mixed methods review [9] in which we used observations from our experience with HIV-infected adults at BMC to define 5 preventable causes of in-hospital mortality associated with HIV infections in sub-Saharan Africa (see Figure 1). We then used narrative review to further characterize each of these barriers and to explored possible interventions which may be used to reduce in-hospital mortality.

## Review

### Late presentation in hospital

#### Observation #1

At BMC we have observed that only 45% of HIV-positive patients admitted to our wards knew their HIV status before admission. The remaining 55% are new HIV diagnoses made in-hospital, and opportunistic infections were the catalyst to obtain HIV testing for almost all of these patients.

Late presentation plays a major role in AIDS related in-hospital deaths in sub-Saharan Africa. Many individuals in sub-Saharan Africa do not access HIV testing and care until they develop opportunistic infections and reach an advanced stage [10]. Table 1 summarizes six studies from sub-Saharan Africa which consistently show patients with HIV/AIDS presenting with a CD4 count <200 and an advanced stage. The median CD4 count of newly diagnosed HIV patients was less than 100 cells/μL. Studies from numerous countries in sub-Saharan Africa have reported similarly high rates of late diagnosis of HIV, or late presentation of HIV infection in hospitals or late presentation for ART treatment.

**Table 1:** **Late presentation to health care facilities of HIV AIDS patients in sub Saharan Africa**

**Reference**	**Country**	**Late stage presentation**	**Patient Population**
Bassett, et al.	South Africa	Median CD4 count of 96 cells/uL	Median CD4 count of patients when deemed ART eligible (not ART initiation) at the Sinikithemba HIV clinic at McCord Hospital.
Mulissa, et al.	Ethiopia	Median CD4 value 156 cells/mm^3^, WHO stage III (49.2%), WHO stage IV (13.3%)	Values of all adult HIV-positive patients at time of presentation to care at Arba Minch Hospital.
Deguenonvo, et al.	Dakar, Senegal (Infectious Diseases Clinic of Fann Teaching Hospital)	Mean CD4 values was 100/mm^3^, CD4 cell lymphocytes ≤ 200 in 86% of cases, AIDS diagnosis in 88% of admitted patients	All HIV-positive patients hospitalized in the Infectious Diseases Clinic of Fann Teaching Hospital in Dakar.
Amuron, et al.	Jinja, Uganda	Median CD4 value was 157/mm^3^ (patients screened between January 6 and December 6, 2005)	HIV-infected subjects being tested for ART eligibility after HIV-diagnosis at The AIDS Support Clinic.
Ingle, et al.	South Africa	Median CD4 value was 101/mm^3^ in 2007	Initial CD4 screening in HIV-infected patients from 36 facilities participating in the Comprehensive HIV and AIDS Management (CHAM) program testing for ART eligibility after HIV diagnosis.
The ART-LINC Collaboration of the International Databases to Evaluate AIDS	Sub-Saharan Africa (Review)	Median baseline* CD4 value was 122 cells/mm^3^ in 2005–2006 in Africa**	HIV-infected patients from numerous health facilities throughout sub-Saharan Africa**.

*Measurements of laboratory values closest to the starting date of ART (within 6 months before up to one week after the date of starting ART) were taken as the baseline levels. For very low CD4 values (<25 cells ⁄ll) and high HIV viral loads (>100 000 copies ⁄ ml) the time window was extended to one year before starting therapy.

**Sites of evaluation from (1) Abidjan, Cote d’Ivoire, (2) Dakar, Senegal, (3) Eldoret, Kenya, (4) Kampala, Uganda, (5) Mbarara, Uganda, (6) Lilongwe, Malawi, (7) Cape Town, South Africa, (8) Johannesburg, South Africa, (9) Soweto, South Africa.

Late presentation has been linked with higher mortality. A study in Brazil recently found that 44.8% of patients who had HIV were diagnosed either during their first hospitalization or less than 30 days prior to hospitalization for an AIDS related illness. For patients diagnosed with HIV at the time of hospitalization from an AIDS related condition, the 12 month survival probability was only 48% [11].

Barriers such as stigma, education, fear of HIV status, long-travel distance to HIV testing sites, lack of confidentiality and delay in test results limit patients’ access to HIV testing [12-19]. Alternative methods of voluntary testing and counseling such as mobile ‘voluntary HIV counseling and testing’ (VCT) clinics and home-based VCT should be employed to increase the number of individuals being tested for HIV. A review by Matovum et al. showed that both alternative methods of VCT can help overcome barriers to early HIV testing [16]. A study by Sweat et al. demonstrated that community mobilization activities can assist in increasing VCT uptake compared to standard clinic-based VCT [20]. HIV testing cannot be limited to a few highly specialized medical centers, but must be made available locally and in a variety of different community-based settings.

### Low rates of in-hospital HIV testing

#### Observation #2

On the wards of BMC, through continuous quality improvement we have been able to improve our HIV testing rates from 10% to 50%. Despite our best efforts, though, we have not been able to consistently achieve HIV testing rates greater than 50%.

Another contributor to in-hospital mortality is a delay or lack of in-hospital HIV testing. To increase the number of individuals who are aware of their HIV serostatus, in 2007 the WHO announced recommendations to offer routine HIV testing in clinical settings now known as ‘provider-initiated testing and counseling’ (PITC). Under the PITC model, providers should recommend testing to every patient who enters a health facility, regardless of the purpose of the visit [21]. Although PITC was unable to achieve 100% HIV testing in the studies identified, research still supports PITC as an effective intervention to increase HIV testing. A controlled trial in Cape Town, South Africa, found that after PITC was implemented, the proportion of patients at the sexually transmitted infections (STI) clinic who received an HIV test increased. Furthermore, PITC made the level of HIV testing more consistent across the sexually transmitted infection (STI) clinics in the area [22]. Another evaluation of PITC in South Africa showed PITC increased HIV testing in patients when compared to voluntary counseling and testing [23]. When PITC was implemented at the Baylor International Pediatric AIDS Initiative (BIPAI) in Tanzania, 81% of all pediatric admissions either tested for HIV or provided valid HIV test results, an increase from only 33.3% with non-routine HIV testing [24]. Lastly, a review of PITC in low- and middle-income countries found that after PITC was implemented, HIV testing increased. Of note, in most studies condom use also increased after PITC. The studies found few negative outcomes, and therefore support PITC implementation and scale-up to increase HIV testing [25]. The authors also concluded that strong leadership was necessary to fully implement the PITC model in high-burden settings.

In some settings, the introduction of PITC has not been successful. PITC was introduced in Tanzania in 2007 after the WHO’s recommendation, but despite PITC, only 10% of all Tanzanians knew their HIV status in 2009 [26]. In Blantyre, Malawi after national recommendations for PITC, prospective analysis showed that of the 18,021 adults who attended the clinic from January to April 2011, only 13.3% were offered HIV testing and counseling [27]. A review of PITC implementation in sub-Saharan Africa by Roura et al. found that the percentage of the target group who were offered an HIV-test by healthcare providers ranged from 24% in an Ethiopian study to 94% in a study from South Africa [28-30].

To assess the barriers to PITC implementation, a cross-sectional study was conducted in 2010 in the government health facilities of Mbeya City Council in Southern Tanzania. All 402 health care providers interviewed had heard of PITC. While the majority of health workers (65.2%) had positive feelings about PITC, 63.2% also felt PITC took too much time and was too involved for them. The majority, 77.9%, of health workers felt PITC should only be offered for patients at high risk of HIV infection, focusing more efforts on STI clinics and symptomatic patients. The most commonly reported obstacle to implementing PITC by health workers was that HIV testing did not seem relevant to the patient’s visit (61%). Other perceived barriers were too few health care workers (42.8%), too many patients (57.7%), lack of PITC training (46.5%), and test kit shortages (37.1%) [31]. Roura, et al. also examined the factors indicated by healthcare providers for not implementing PITC. The two main points were fear of offending patients by offering HIV testing and a perceived slowing of patient care in the clinic [28].

In examining the impact of PITC on limited human resources, one study in Kenya found that implementing PITC increased the median consultation time from 10 to 17 minutes [32]. Another study in Zambia showed that patient-provider contact time increased by 55% after PITC was implemented [33]. Several areas have tried to address the impact of PITC on human resources by using trained lay counselors and assistant physicians to conduct PITC [33,34]. A group in South Africa tried to optimize human resources by conducting group pre-test counseling sessions (keeping the option of individual sessions if desired by patients) [35]. More research needs to be conducted on how to use existing human resources to efficiently and effectively implement PITC in-hospitals with the current human resources and to overcome perceived and real barriers that prevent health care workers from fully implementing this procedure.

### Laboratory capacity and delayed OI diagnosis and treatment

#### Observation #3

At BMC, despite the fact that CD4 count is requested for all HIV patients and is required for initiation of ART in a patient, less than 20% of patients receive results during hospitalization, and the turnaround time is an average of five days.

Low laboratory capacity was also identified as a cause of in-hospital mortality, leading to delayed CD4 testing and OI diagnosis and treatment. CD4 testing is important for patient care in order to monitor patients and to determine who is eligible to initiate ART, but it requires a flow cytometer which is not routinely available in many laboratories in the developing world [36]. Furthermore, even when available many existing laboratory instruments end up non-functional or without reagents for testing [37]. For example, between 2009 and 2010 in Malawi, only 52 of the 396 ART sites (13%) had CD4 flow cytometers, and of the 52 machines, 10 (20%) of the CD4 machines in the country were not functional [38]. Technologically, CD4 machines are complex, requiring regular maintenance, a steady stock of reagents, and skilled staff [36]. Laboratory staff shortages for CD4 testing become even more apparent when VCT or PICT interventions are scaled up in high-prevalence contexts. The number of identified HIV patients in need of CD4 tests increases and strains the already limited laboratory resources [36]. Advances in point-of-care CD4 testing with simplified technology offer an opportunity to both expand access and increase the speed of CD4 tests.

As noted, low laboratory capacity also leads to delays in OI diagnosis and treatment. A review of tuberculosis, a common opportunistic infection, demonstrates these delays. Studies in Uganda, Botswana, Malaysia and Ethiopia found median health system delays for TB diagnosis ranging from 4 to 12 weeks [39-43]. One study in Nairobi, Kenya, showed a very high level of under-diagnosis of smear positive tuberculosis; of individuals who had presumed smear negative pulmonary tuberculosis, 26% had smear positive results when re-examined carefully with repeat sputum smears [44]. In our experience in Tanzania, delays in collecting sputum samples in hospitalized patients and then retrieving lab results can cause a 7–10 day delay between admission and diagnosis of smear-positive tuberculosis. Delays in diagnosis and treatment for tuberculosis lead to increased morbidity and mortality [39]. Though little research has been done on diagnosis and treatment delays of other opportunistic infections in resource-poor settings, we believe tuberculosis is a good example of the delays that may be happening in the diagnosis and treatment of other opportunistic infections.

Shortage of laboratory personnel, limited funding, and limited technical support are some reasons noted for constraining expansion of necessary diagnostic testing and limiting laboratory capacity in sub-Saharan Africa [45,46]. The severe shortage of laboratory personnel in many developing countries is the result of limited professional training and accreditation programs [45]. Although accurate diagnostic tools are necessary to monitor HIV/AIDS progression and opportunistic infections, laboratory development has not received the same level of priority in funding as clinic activities [46].

In 2010, the WHO of the African region recognized the need for laboratory capacity building, and outlined a series of actions that need to be taken to bolster laboratory capacity in Africa. This includes the development of a comprehensive national laboratory policy with a national laboratory strategic plan, laboratory leadership, funding sources, national reference laboratory, laboratory supply and distribution systems, and quality assurance [47].

### Delay in initiation of ART in-hospital

#### Observation #4

At BMC, even though most HIV-positive hospitalized patients at BMC may qualify to begin ART either based on CD4 counts (mean CD4 count of 248 cells/mm3) or presence of an opportunistic infections like tuberculosis, still <1% of HIV positive patients are initiated on ART in the wards.

Another preventable health system failure that contributes to HIV-related in-hospital deaths is the delay in initiating ART. Early ART initiation in AIDS patients is important for survival. Bekker et. al noted that if an individual who is first diagnosed as HIV-positive is already at the AIDS stage of the disease, the ART eligibility threshold has previously been crossed. The review then found that each ensuing month’s delay in initiating ART treatment after presenting in a health system with an AIDS diagnosis increases mortality 4–8% [10].

Another randomized trial performed in Haiti found that early ART-initiation (>200 and <350 CD4 T-cells per mm3) compared to ART initiation at a CD4 T-cell count <200 decreased the rates of death by 75% and incident tuberculosis by 50%, leading the WHO to revise ART treatment initiation guidelines from 200 to 350 cells/mm [48]. Overall, prompt ART initiation once HIV status and ART eligibility are determined increases life expectancy compared to delayed ART initiation [10,48-51].

Numerous studies have documented the impact of early initiation of ART in newly diagnosed HIV-infected patients who present with tuberculosis. Findings in one study in South Africa showed that the incidence of death was significantly less in patients with a CD4 count less than 50 cells/mm^3^ when ART treatment was started within 4 weeks of the initiation of tuberculosis treatment versus starting ART later (within 4 weeks of the continuation phase of TB treatment) [52]. Another study by Havlir, et al. found that for individuals with CD4+ counts less than 50 cells/mm^3^, immediate ART initiation (within 2 weeks of starting TB therapy) led to 42% less AIDS illnesses or deaths than ART initiation 8–12 weeks after TB treatment initiated [53]. Similarly, in Cambodia, Blanc, et al. found that patients with a CD4 count less than 200 cells/mm^3^ who started ART within 2 weeks of TB treatment initiation experienced significantly better survival rates than those who started ART 8 weeks after TB treatment initiation [54].

In undiagnosed patients presenting in hospital with OIs, initiating ART during OI treatment resulted in better outcomes than waiting to begin ART. Zolopa, et al. found that patients who began ART during OI treatment had significantly lower levels of AIDS progression and death than the patients that waited until completing OI treatmentto begin ART [55]. More specifically, this study demonstrated that in a cohort of very sick HIV-infected patients that presented to hospitals with OIs and a mean CD4 count of 47 cells/mm^3^, initiating ART simultaneously with OI treatment significantly decreased death and AIDS progression versus waiting 1 month after OI treatment initiation to begin ART [55,56].

Though early ART initiation has been proven to have positive impacts on patient outcomes, there is a lack of integration between HIV testing and initiation in hospitals. The lack of integration was illustrated in a retrospective study from Kamuzu Central Hospital in Malawi. Although over 85% of patients identified as HIV-infected were already ART-eligible during hospitalization, none started ART in hospital [24].

### Loss to follow-up (LTFU) upon discharge from hospital

#### Observation #5

At BMC, the lack of integration of HIV clinics to primary care services has made follow-up of these patients after discharge from hospital difficult. Our patients stay in the hospital an average of one week, and on discharge are advised to attend the nearest HIV clinic of their choice. Due to mixed feelings such as denial and stigma associated with HIV, many patients delay seeking help at HIV clinics and hence re-present to hospitals only when they develop new or recurrent opportunistic infections.

Many HIV-infected individuals are lost to follow up after their hospital stay. Patients lost to follow-up have higher mortality than those who remain in an HIV care and treatment program, and may re-present to hospitals in the future at an advanced disease stage with high rates of mortality. Data collected through the National Care and Treatment database in Tanzania was used to analyze HIV care and LTFU in 88,875 adults from 101 clinics in Tanzania. This analysis found that 12 months after ART treatment initiation, 18% of adults had already been lost to follow-up. After 36 months, 36% had been lost to follow-up [57]. Another review of patient retention in sub-Saharan Africa found that after 2 years of treatment, only 60% of patients were retained in HIV ART care [58].

Such failure or delay in outpatient care linkage is widespread and has been well documented. In Ethiopia, reports from the Ministry of Health from 2005 to 2008 showed that only 61% of patients newly diagnosed with HIV were linked to chronic care services [59]. In Gugulethu outpatient ART program in Cape Town, 66% of deaths actually occurred within 90 days of pre-ART enrollment in the patients who were awaiting ART initiation. Overall, this study demonstrated that the health system failure resulting in a 3-month delay of ART led to a substantial number of deaths in patients with severe immune suppression [60].

Exact causes of LTFU are often difficult to understand and not well documented. A review of patients LTFU in ART-treatment programs in resource-limited settings found that 83% of losses were due to an incomplete, incorrect, or missing telephone number or address. Another 16% of patients had moved from the address in the patient file to an unknown location or distant location. Among LTFU patients who were found alive, reasons frequently cited for leaving the ART programs were transfer to another program, cost of transport (financial problems), and improved or deteriorating health. Less common answers included stigma and social problems and adverse events. In LTFU patients who died, 63% had unrecorded causes of death, while another 23% died from AIDS defining illnesses [61].

A cost analysis of preventing loss to follow up examined four specific interventions, with each subsequent intervention increasing the costs. First, ART should be provided for free to patients. The second incremental intervention also eliminates the cost of patients for medications that treat opportunistic infections. The third intervention aims to improve HIV care skills among health workers. The final intervention provides patients with food during the day of visits for ART and cost of transportation. The study found that these interventions, if implemented with a success rate of retaining at least 12–41%, would be cost effective under when analyzed using the Cost-Effectiveness of Preventing AIDS Complications (CEPAC) International model [62].

## Conclusions

This review explored the potential causes of preventable in-hospital mortality associated with HIV infections in sub-Saharan Africa in the anti-retroviral era. Based on our experience as healthcare providers in Africa and a review of the literature we identified 5 health systems failures which may cause preventable in-hospital mortality, including late presentation of HIV cases, low rates of in-hospital HIV testing, poor laboratory capacity which limits CD4 T-cell testing and the diagnosis of opportunistic infections, delay in initiation of ART in-hospital, and lastly, problems associated with loss to follow-up upon discharge from hospital. Using our findings, along with the current available literature, possible interventions should be discussed and developed which may be used to reduce in-hospital mortality.

## Abbreviations

AIDS: Acquired immunodeficiency syndrome
ART: Anti-retroviral therapy
BIPAI: Baylor International Pediatric AIDS Initiative
BMC: Bugando Medical Centre
CEPAC: Cost Effectiveness of Preventing AIDS Complications
HIV: Human immunodeficiency virus
LFTU: Loss to follow-up
OI: Opportunistic infection
PEPFAR: The US President’s Emergency Plan for AIDS Relief
PITC: Provider-initated testing and counseling
STI: Sexually transmitted infection
TB: Tuberculosis
UNGASS: United Nations General Assembly Special Sessions
US: United States
VCT: Voluntary HIV counseling and testing
WHO: World Health Organization
